# Brazilian cross-cultural adaptation of “Return-to-work self-efficacy” questionnaire

**DOI:** 10.1590/S1518-8787.2017051006778

**Published:** 2017-02-23

**Authors:** João Silvestre Silva, Rosane Härter Griep, Suzanne E Lagerveld, Frida Marina Fischer

**Affiliations:** I Programa de Pós-Graduação em Saúde Pública. Faculdade de Saúde Pública. Universidade de São Paulo. São Paulo, SP, Brasil; II Gerência Executiva São Paulo Norte. Instituto Nacional do Seguro Social. Ministério do Desenvolvimento Social e Agrário. São Paulo, SP, Brasil; III Laboratório de Educação em Ambiente e Saúde. Instituto Oswaldo Cruz. Fundação Oswaldo Cruz. Rio de Janeiro, RJ, Brasil; IV Amsterdam University of Applied Sciences. Amsterdam, The Netherlands; V Department of Social and Organizational Psychology. Utrecht University. Utrecht, The Netherlands; VI Departamento de Saúde Ambiental. Faculdade de Saúde Pública. Universidade de São Paulo. São Paulo, SP, Brasil

**Keywords:** Occupational Health, Mental Disorders, Sick Leave, Return to Work, Occupational Medicine, Surveys and Questionnaires, Translations

## Abstract

**OBJECTIVE:**

To describe the translation and early stages of cross-cultural adaptation of the questionnaire *Verwachtingen over werken* (or “Return-to-work self-efficacy”) for workers in sick leave due to mental disorders, from the original in Dutch to the Brazilian Portuguese language.

**METHODS:**

A panel gathering experts was formed to determine the questionnaire conceptual and item equivalence. For semantic equivalence, the Dutch-Portuguese Brazilian translations were consolidated and consensus meetings were held to structure versions of the instrument. Each version was back-translated from Brazilian Portuguese to Dutch and evaluated by one of the authors of the original version. The final version was submitted to two pre-tests for operational equivalence.

**RESULTS:**

The original questionnaire in Dutch was translated twice to Brazilian Portuguese. During the process, four consensus meetings of the experts’ panel were performed to create the versions. Each version was back-translated to Dutch. One of the authors of the original questionnaire performed an evaluation on the first three versions until the definition of the final one, which was titled *Expectativas sobre o trabalho* (Expectations about work). Pre-tests’ participants did not reported problems to fill the questionnaire.

**CONCLUSIONS:**

Results indicate that the Brazilian Portuguese cross-culturally adapted version maintains the original meaning of the questionnaire, while including characteristics peculiar to the Brazilian reality. Measurement and functional equivalence of this version must still be evaluated before its application can be recommended for workers who have been absent from work due to mental disorders.

## INTRODUCTION

Impact of sick leaves on social security expenditures is a global problem^13^. Within this context, sick leaves due to mental disorders are known to have the longest duration^5^and are a source of significant social security expenditures^4^. In Brazil, mental diseases are the third main cause for social security sickness benefits, which are paid to ensured workers from the 16th day of sick leave^14^.

Identification and characterization of groups at high risk for long term sick leave are necessary to develop indicators for programs to facilitate return to work. Professionals involved in these programs should approach workers in sick leave as early as possible to maximize the reintegration to workplace likelihood^12^. Unfortunately, there is not official guideline to support professionals about return-to-work or validated questionnaire that assesses if workers are at risk for long term sick leave in Brazilian reality.

Among the many determinants involved in the return to work process, the self-efficacy is one of the relevant individual parameters studied in cases of disability due to mental disorders^8-10,16^. Self-efficacy refers to an individuals’ belief in his or her capacity to execute tasks and behaviors in a satisfactory manner^1,2^. This concept guided the construction of two instruments to assess the workers’ perception of their ability to perform their usual tasks upon returning to work after sick leaves due to musculoskeletal^3^ and mental disorders^9^. Although both instruments were named “Return-to-work self-efficacy” (RTW-SE), only the former was validated for English-speaking populations. The latter was elaborated in Dutch *(Verwachtingen over werken*) and validated in three groups of workers in the Netherlands.

In the Dutch questionnaire, workers with mental disorders are requested to imagine themselves going to work the following day and to describe their expectations (as a function of their ongoing emotional state and state of mind). The questionnaire includes 11 statements, and respondents are requested to manifest their degree of agreement or disagreement with them on a six-point Likert scale. The sum of the points attributed to each item provides an individual score; the average score of the studied group is considered the cutoff point to dichotomize the perceived return-to-work self-efficacy as low or high. The construct validity of the RTW-SE version validated for workers with mental disorders was considered satisfactory as a function of its good correlation with the dimensions included in the theoretical model: perceived general self-efficacy, locus of control, coping, physical workload, and mental health problems. The questionnaire exhibited excellent internal consistency and adequate test-retest reliability, proved to be sensitive to changes over time, and behaved as a robust predictor of return to work within three months^9^.

In studies conducted in the Netherlands, RTW-SE demonstrated ability to predict the time for^8,10,16^ and success in actual return to work^9^. Thus, application of RTW-SE to Brazilian workers in sick leave due to mental disorders might be useful to detect situations in which having low self-efficacy is an indicator of difficulties to return to work.

The aim of the present study was to perform the translation and early stages of cross-cultural adaptation of RTW-SE questionnaire for workers in sick leave due to mental disorders from the original Dutch to the Brazilian Portuguese language.

## METHODS

The translation and cross-cultural adaptation of RTW-SE were performed according to the methods indicated in the scientific literature^6,7,11^. Efforts were made to maintain the concepts present in the Dutch version, while adapting them to Brazilian cultural equivalents. Conceptual, item, semantic, and operational equivalence were determined. The evaluation of the measurement and functional equivalence will be described in future studies, since they are currently being tested in a multicenter study.

Conceptual equivalence is achieved when the concepts included in the original and adapted questionnaires have similar meaning and equally affect respondents from both cultures^7^. To test conceptual equivalence between the Dutch and Brazilian versions of RTW-SE we performed a review of the literature on the subject “return to work after sick leave due to mental disorders”. In that stage of the study, our aim was to establish the relevance of the core subject of the questionnaire for the Brazilian reality. For this purpose, a panel of experts with large experience in the conduction of studies on absenteeism by disease was formed, which included one full professor in public health (FMF), one occupational epidemiologist (RHG), and one specialist in occupational medicine and social security legislation (JSSJ). Item equivalence was analyzed in one of the panel meetings to establish the adequacy and clarity of the statements and response options.

Semantic equivalence is concerned with the transfer of the meaning of concepts by words and sentences of different languages to equally affect respondents of different cultures^7^. This aspect presented some difficulty, since the questionnaire was presented in English in the article describing the validation study^9^, while the version actually applied in that study was the original Dutch one. To overcome that problem, we performed the following steps, which are schematically described in Figure:

**Figure:**
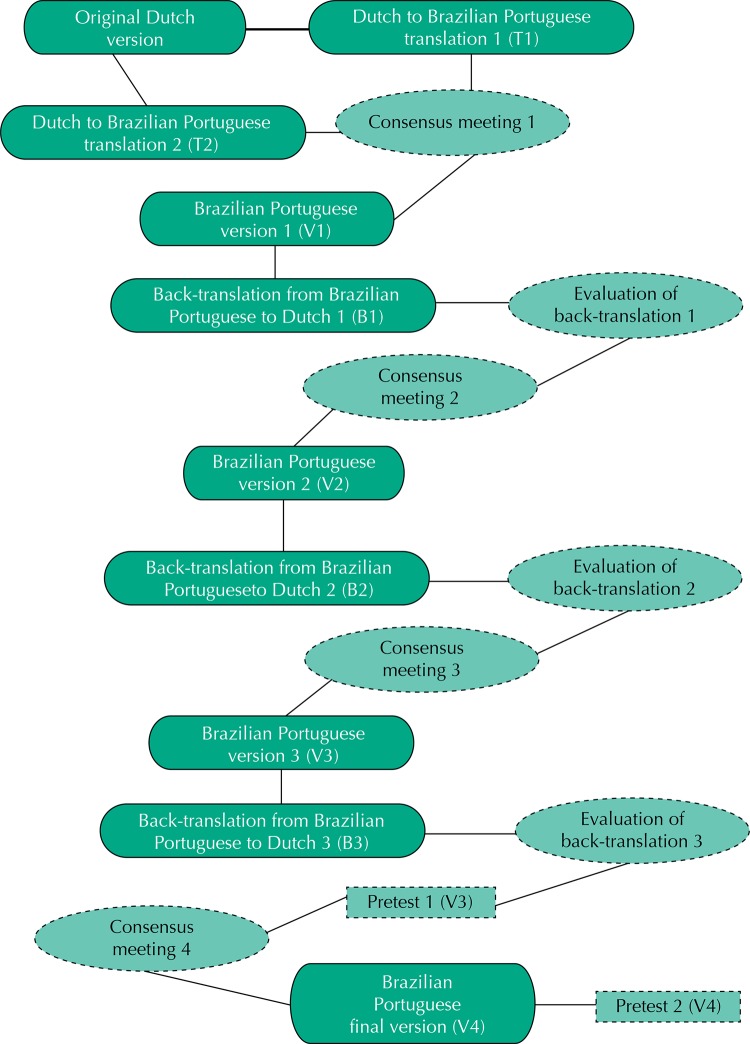
Flowchart representing the process of cross-cultural adaptation of the questionnaire “Return-to-work self-efficacy” (RTW-SE) for workers with mental disorders.

1. Translation: the Dutch version of the questionnaire was split into 22 sentences, as follows: name (one sentence), questionnaire introductory remarks (three sentences), orienting sentence (one sentence), statements (11 sentences), and Likert scale (six sentences). An international certified translation service was hired to independently perform two translations from Dutch to the Brazilian Portuguese language (T1 and T2);
2. First consensus meeting: panel members assessed the clarity of both translations, the use of colloquial language, and the semantic equivalence between items. On those grounds, changes were made, resulting in the first Brazilian Portuguese version (V1);
3. Back-translation: the Brazilian Portuguese version was back-translated to Dutch (B1). Next, one of the original authors of the questionnaire (SEL) was requested to grade the concordance between the original Dutch and back-translated versions from zero to 10. Whenever a sentence scored less than 10, the Dutch reviewer should describe in detail the divergences between versions;
4. Second and third consensus meetings: a second consensus meeting was held after the evaluation of B1 to discuss the need for changes and to elaborate a new Brazilian Portuguese version (V2). This new version was back-translated to Dutch (B2). The procedure was repeated one further time (third consensus meeting → V3 → B3) until the final Brazilian Portuguese version was achieved. We had previously established that the final version was to be the one in which no sentence was graded below 8.5 (eight and a half) by the back-translation reviewer.

Two pretests were performed with individuals in sick leave due to mental disorders to assess the operational equivalence between the questionnaire versions. The aim of the first pretest was to establish whether words and expressions were adequate to the Brazilian reality and verbal constructions clear enough and appropriate for Brazilian Portuguese speakers. In the second pretest, the final version was applied to 17 individuals to investigate whether they had any difficulty in understanding the questionnaire content and responding it. Experts then held the fourth consensus meeting, in which they decided that the version used in the second pretest represented the cross-cultural adaptation of RTW-SE for workers with mental disorders to the Brazilian Portuguese language.

The present study was approved by the human research ethics committee of Faculdade de Saúde Pública, Universidade de São Paulo, Brazil (Certificate of Presentation for Ethical Appraisal – CAAE 23492013.5.0000.5421).

## RESULTS

The experts panel considered that domains described in the scale validation study^9^ could be of interest for the Brazilian reality and that they agreed with the local occupational and social security conditions. The first topic discussed by the panel of experts was the questionnaire name. While the literal translation of the Dutch name is “Expectations about work”, in the validation study the questionnaire was named “Return-to-work self-efficacy” in English. The experts agreed that terms “efficacy” or “self-efficacy” misrepresented the original name, in addition to the fact that word “expectations” is much more widely used in Brazil than “self-efficacy”. Moreover, it is worth to notice that the English version of the questionnaire has not yet been validated.

The questionnaire name changed from V1 to V2. The name initially suggested was “Expectations about return to work”; however, as a function of comments made to the back-translation, the experts observed that the questionnaire can be usefully applied not only to workers in sick leave, but also to the ones who already returned to work. In the latter case, the aim of the questionnaire application is to monitor changes in the expectations of workers in process of returning to work. Therefore, the selected name was “Expectations about work”.

In Brazil, public and private organizations are entitled to reduce the working hours and workload, but a proportional reduction in the salary is illegal. However, the ongoing trend is for employers allow employees returning to work after a sick leave to shift to part-time employment. The contradiction between labor law and the perception of the panel of experts about what really happens in practice resulted in the need to discuss the questionnaire’s introductory remarks. The original Dutch version comprises three options: if the worker is currently not working, or partially returned to work, or fully returned to work. Considering the possibility of part-time work, especially in companies that allow for gradual return to work, we chose not to make any changes in the original introductory remarks.

Still in this regard, the back-translation reviewer observed that V1 overemphasized the mandatory aspect of return to work (“Imagine that you have to return to work tomorrow…”). Therefore, the final version states: “Imagine that you’re returning to work tomorrow (in your present state of health)”.

Also, the orienting sentence had to be rephrased following the evaluation of the first back-translation. For the same reasons mentioned above, it was changed to emphasize the possibility aspect (“If I were to return to work tomorrow…”).

Concerning the end of the orienting sentence, the experts discussed whether to keep the active verb “to expect” as in the original Dutch version (“If I were to return to work tomorrow I expect…”) or use the noun “expectations” as in the questionnaire name. In Portuguese, the latter formula (“… my expectations are…”) is more akin to the structure of the questionnaire statements, which begin by a verb in infinitive form (“to be able to”, “not to be able to”, “not to have” etc.) and provides a more accurate perspective of the immediate future.

As concerns statement 1, the two initial translations included words seldom used in colloquial Brazilian Portuguese (“*contratempos/adversidades*”: setbacks). The panel of experts suggested using “difficulties” or “problems” instead; the evaluation of the first back-translation indicated that the word “difficulties” better matched the statement’s intention.

Statements 2, 4, 5, and 6 did not pose any problems requiring special attention along the process of cross-cultural adaptation of the questionnaire, since the translations successfully represented the concepts meant in the original Dutch version, according to the back-translation reviewer.

Statement 3 (“I will be able to set my personal boundaries at work”) was a subject of much discussion at the first consensus meeting, because its essential meaning was not clear. In one of the translations, the corresponding expectation was described as of being able to control work tasks, while in the other, as of being able to set boundaries to work pressure. Following consultation to the authors of the original questionnaire, the experts understood that statement 3 concerns the workers’ awareness of their individual boundaries to the performance of work tasks and their expectation of being able to avoid overstepping them.

Regarding statement 7, the problem was the best word to qualify the workers’ ability to concentrate on their work. Although the adverb “well” (“*bem*”) is commonly used in Brazil, the back-translation with the highest score was the one that used the word “enough” (“*suficiente*”) instead. The back-translation reviewer considered the latter was less emphatic and thus closer to the original meaning.

As in the case of statement 3, the focus of the discussion regarding statement 8 was also the essential meaning of the corresponding expectation. According to one translation, it concerned the ability to cope with the pressure exerted by interpersonal relationships at work (with colleagues, supervisors, customers), while the other translation is about the discomfort caused by the organization of the work environment (hustle and bustle). The panel of experts agreed that the latter was the most adequate.

Concerning statement 9, the discussion centered on the choice of the most adequate verb; the options were “to handle” (“*lidar*”) or “to solve” (“*resolver*”) possible problems at work. The back-translation reviewer considered the latter to be the closest to the original Dutch version.

As for statement 10, the experts understood that the best manner to assess the motivation to work is by motivating behavior rather than based on transient motivational states or being motivated by extrinsic influences.

In statement 11, the verb “to meet” (“*cumprir*”) was considered more adequate than “to deal” (“*lidar/gerir/gerenciar*”) with the physical demands of work.

The adaptation of the six-point Likert scale did not pose any problem.

In the evaluation of the first back-translation, the average score attributed by one of the authors of the original Dutch questionnaire to the first Brazilian Portuguese version (V1) was 7.89 (total score corresponding to the sum of 22 items = 173.5). At the second consensus meeting, the panel of experts made changes in all items, except for the Likert scale (which had been attributed the maximum score), resulting in the second Brazilian Portuguese version (V2). The average score attributed by the Dutch reviewer to the second back-translation (B2) was 9.34 (total score for 22 items = 205.5).

During the third consensus meeting, experts revised four out of the 22 items and established the third Brazilian Portuguese version (V3). The average score attributed by the Dutch reviewer to the corresponding back-translation (B3) was 9.66 (total score for 22 items = 212.5).

This version (V3) was used in the first pretest. With the follow-up, the experts discussed in the fourth consensus meeting chose to add the expression “at/of/in work” (“*no trabalho*”, “*do trabalho*”, “*durante o trabalho*”) in statements 2, 4, 5 and 6 to reinforce the fact that the questionnaire focuses on the respondents’ expectations regarding work situations upon returning to it after a sick leave. This change might be considered as a cross-cultural adaptation performed after a pretest with the target population.

Following these modifications in V3, the new version (V4) was considered to be adequate as the final one by all the authors (Table).

**Table:** Original Dutch version and cross-cultural adaptation to the Brazilian Portuguese language of the questionnaire “Return-to-work self-efficacy” (*Verwachtingen over werken*) for workers in sick leave due to mental disorders.

Version	Dutch original	Brazilian Portuguese final version
Name	*Verwachtingen over werken*	*Expectativas sobre o trabalho*
Introductory remark (sentence 1)	*De volgende stellingen hebben betrekking op uw verwachtingen over werken.*	*As afirmativas a seguir dizem respeito às suas expectativas sobre o trabalho.*
Introductory remark (sentence 2)	*Het kan zijn dat u momenteel nog helemaal niet aan het werk bent, of dat u gedeeltelijk aan het werk bent, of misschien bent u al weer volledig aan het werk.*	*Pode ser que você atualmente não esteja trabalhando, ou que você esteja trabalhando em tempo parcial, ou ainda que você já tenha retomado integralmente ao trabalho.*
Introductory remark (sentence 3)	*Stelt u zich de situatie voor dat u morgen volledig aan het werk bent of gaat (met uw huidige klachtenniveau).*	*Imagine que você retorne ao trabalho amanhã (com a sua condição de saúde atual).*
Orienting sentence	*Als ik morgen weer volledig aan het werk zou gaan, dan verwacht ik dat:*	*Se eu retornar ao trabalho amanhã, minha expectativa é:*
Statement 1	*Ik tegenslagen goed aan kan pakken.*	*Ser capaz de lidar bem com as dificuldades no trabalho.*
Statement 2	Ik door mijn emoties mijn taken niet goed kan uitvoeren.*	Não conseguir executar bem minhas tarefas de trabalho por causa do meu estado emocional.*
Statement 3	*Ik in staat ben mijn grenzen te bewaken.*	*Ser capaz de estabelecer limites na realização das tarefas de trabalho.*
Statement 4	*Ik mijn taken uit kan voeren.*	*Conseguir executar minhas tarefas no trabalho.*
Statement 5	*Ik met emotioneel veeleisende situaties om kan gaan.*	*Conseguir lidar com situações emocionalmente difíceis no trabalho.*
Statement 6	Ik geen energie meer over zal hebben voor iets anders.*	Não ter mais energia para fazer qualquer outra coisa.*
Statement 7	*Ik mij voldoende kan concentreren op mijn werk.*	*Conseguir me concentrar o suficiente no meu trabalho.*
Statement 8	*Ik de drukte op het werk weer aankan.*	*Conseguir lidar com a agitação no trabalho.*
Statement 9	Ik mogelijke problemen op mijn werk niet kan oplossen.*	Não conseguir resolver os possíveis problemas no trabalho.*
Statement 10	*Ik mezelf voldoende kan motiveren om mijn werk te doen.*	*Motivar-me o suficiente para realizar o meu trabalho*
Statement 11	*Ik aan de fysieke eisen van mijn werk kan voldoen.*	*Conseguir cumprir com as exigências físicas do meu trabalho.*
Likert scale (sentence 1)	*Helemaal oneens*	*Discordo totalmente*
Likert scale (sentence 2)	*Groten-deels oneens*	*Discordo em grande parte*
Likert scale (sentence 3)	*Beetje oneens*	*Discordo um pouco*
Likert scale (sentence 4)	*Beetje eens*	*Concordo um pouco*
Likert scale (sentence 5)	*Groten-deels eens*	*Concordo em grande parte*
Likert scale (sentence 6)	*Helemaal eens*	*Concordo totalmente*

* reverse score.

The individuals invited to respond the final version of the questionnaire did not report any difficulty in understanding or filling in it. Most of the second pretest participants were female (66.7%), education equal or over 11 years (80.0%), and diagnosis of depressive disorder (60.0%). Their mean age was 36 years (SD ± 7.08 years).

## DISCUSSION

Comparison between the original questionnaire and the back-translation of the third Brazilian Portuguese version (V3) showed that the referential meaning of the questionnaire name, introductory remarks, orienting sentence, and Likert scale was similar between both. Inclusion of expression “at/of/in work” at a few statements in the final Brazilian Portuguese version (V4) might be considered a small change concerning the original questionnaire. Thus, the cross-adaptation of the instrument succeeded in maintaining its original meaning, while including characteristics peculiar to the Brazilian reality.

Since workers’ self-efficacy might be a predictor of the time for and success of return to work^8-10,16^, knowledge of the workers’ expectations might allow detecting difficulties in this regard. Compared with simple and short questions about expectations, the RTW-SE scale seems better suited to predict the actual return-to-work process^9^. As the original version of the questionnaire exhibited satisfactory internal consistency and sensitivity over time^9^, its application might be useful to health care professionals, occupational support team, and social security services by providing ideas to improve workers’ self-efficacy for return to work.

The social exclusion of ill workers might interfere with individual, economic, and psychosomatic aspects of their lives. Long sick leaves perpetuate the workers’ suffering, which certainly does not help to improve their physical and mental wellbeing^15^. Application of questionnaire “Expectations about work” to Brazilian workers might contribute to the planning of return-to-work procedures for individuals with disabling mental disorders.

Knowledge on the workers’ limitations about returning to work provided by the questionnaire might further contribute to the formulation of public (e.g., vocational rehabilitation improvement) and private policies (e.g., guidelines to improve the return-to-work process). It might also be useful for occupational health professionals to achieve an adaptation point between work demands and employees’ ability, whereas they are returning from sick leave due to mental disorders. Thus, the use of the questionnaire by professionals might exert positive influence on the social reinsertion of workers and contribute to monitoring the efficiency of the whole process.

In conclusion, the present article describes the initial steps in the cross-cultural adaptation of the questionnaire “Return-to-work self-efficacy” after sick leave due to mental disorders to the Brazilian Portuguese language. Measurement and functional equivalence of the adapted version must still be assessed before its application can be recommended. Future studies ought to assess the scientific validity of the Brazilian Portuguese version in such a way to be used in research as well in private and public occupational health services.
